# Pseudoginsengenin DQ Exhibits Therapeutic Effects in Cisplatin-Induced Acute Kidney Injury via Sirt1/NF-κB and Caspase Signaling Pathway without Compromising Its Antitumor Activity in Mice

**DOI:** 10.3390/molecules23113038

**Published:** 2018-11-21

**Authors:** Zeng Qi, Zhuo Li, Wei Li, Yunhe Liu, Cuizhu Wang, Hongqiang Lin, Jinping Liu, Pingya Li

**Affiliations:** 1 School of Pharmaceutical Sciences, Jilin University, Changchun 130021, China; qizeng95@163.com (Z.Q.); zhuoli198602@gmail.com (Z.L.); craneliu30@gmail.com (Y.L.); wangcz15@mails.jlu.edu.cn (C.W.); linhq17@mails.jlu.edu.cn (H.L.); liujp@jlu.edu.cn (J.L.); 2 College of Chinese Medicinal Materials, Jilin Agricultural University, Changchun 130118, China; liwei7727@126.com

**Keywords:** pseudoginsengenin DQ, CDDP-induced acute kidney injury, Sirt1/NF-κB, caspase

## Abstract

In this study, the protective effects of pseudoginsengenin DQ (PDQ) on cisplatin (CDDP)-induced nephrotoxicity were assessed, with a primary investigation into the mechanisms involved. Our results showed that pretreatment with PDQ remarkably restored levels of blood urea nitrogen (BUN) and creatinine (CRE), malondialdehyde (MDA), glutathione (GSH), superoxide dismutase (SOD), catalase (CAT), tumor necrosis factor-*α* (TNF-*α*), and interleukin-1*β* (IL-1*β*). Meanwhile, PDQ decreased the CDDP-induced overexpression of heme oxygenase 1 (HO-1), cytochrome P450 E1 (CYP2E1), TNF-*α*, nuclear factor-kappa B (NF-κB), cyclooxygenase-2 (COX-2), and inducible nitric oxide synthase (iNOS) in renal tissues. Hoechst 33258 and TdT-mediated dUTP nick-end labeling (TUNEL) staining showed that CDDP-induced renal tubular cell apoptosis was apparently inhibited by PDQ. Western blotting showed that PDQ reversed the CDDP-induced (1) downregulation of Sirtuin-1 (Sirt-1), nuclear-related factor 2 (Nrf2), and Bcl-2, and (2) upregulation of NF-κB, Nox-4, Bax, caspase-9, and caspase-3. In addition, PDQ enhanced the antitumor activity of cisplatin in Lewis lung cancer xenograft tumor model mice. In conclusion, we found that PDQ exerted a renal protective effect against CDDP-induced acute nephrotoxicity via Sirt1/NF-κB and the caspase signaling pathway without compromising the antitumor activity of CDDP, which provides a new potential strategy for the clinical treatment of cancer and presents a new medicinal application of PDQ.

## 1. Introduction

Cisplatin (CDDP), an inorganic platinum antitumor agent, is widely used in clinical chemotherapy for the treatment of a variety of human tumors, including ovary, cervical, testicular, non-small cell lung cancer, and other malignancies [1]. However, the frequency of life-threatening CDDP-induced acute kidney injury (AKI) is a serious and unneglectable clinical problem [2]. The nephrotoxicity of CDDP lessens the willingness of patients and healthcare providers to consider this therapy except under extreme circumstances and leads to the limitation of its clinical applications [3]. Therefore, there is an urgency to find effective therapeutic regimens to reduce CDDP-induced nephrotoxicity in the treatment of malignancies for the benefit of patients.

Pseudoginsengenin DQ (pseudoginsengenin of diol derivatives quest, PDQ), the product of the oxidative cyclization of protopanaxadiol, exhibits a significant pharmacological effect as an antiarrhythmic agent [4]. According to a previously published report, PDQ could attenuate isoproterenol-induced myocardial ischemia injury by inhibiting the increase of malondialdehyde content, the reduction of superoxide dismutase, and the activity of glutathione peroxidase [5]. However, the effects of PDQ on CDDP-induced AKI are still unknown. 

Apoptosis is an important factor in the pathogenesis of CDDP-induced AKI [6,7]. Under normal circumstances, apoptosis is a permanent and fundamental form of programmed cell death that occurs in multicellular organisms [8]. Although apoptosis is a regular biological phenomenon, acute cellular injury also can trigger defective apoptosis and further touch off tissue necrosis in CDDP-induced AKI [9]. It was reported that oxidative stress and inflammation are also leading factors in CDDP-induced nephrotoxicity [10]. As CDDP exposure causes renal dysfunction, accompanying oxygen-derived free radicals (ROS) lead to oxidative stress, such as excessive lipid peroxidation, which results in a response of numerous proinflammatory cytokines and inflicts irreversible cellular damage in the kidney [11,12,13]. Sirtuin-1 (Sirt-1) is an important protein that is involved in the biological processes of CDDP-induced oxidative stress and inflammation by activating transcriptional activity of downstream genes, including nuclear-related factor 2 (Nrf2) and nuclear factor-kappa B (NF-κB) [14,15]. Thus, Sirt-1 regulates the formation of ROS and development of oxidative stress, and affects the levels of proinflammatory cytokines and inflammatory mediators, such as interleukin-1*β* (IL-1*β*) and tumor necrosis factor-*α* (TNF-*α*), resulting in a severe inflammatory reaction in the kidney [16,17].

In this study, the protective effects of PDQ against CDDP-induced AKI, as well as the molecular mechanisms of PDQ, were investigated by examining its involvement in antioxidant, anti-inflammatory, and anti-apoptotic activity. 

## 2. Results

### 2.1. PDQ Protects against CDDP-Induced Renal Dysfunction

**Figure 1:**
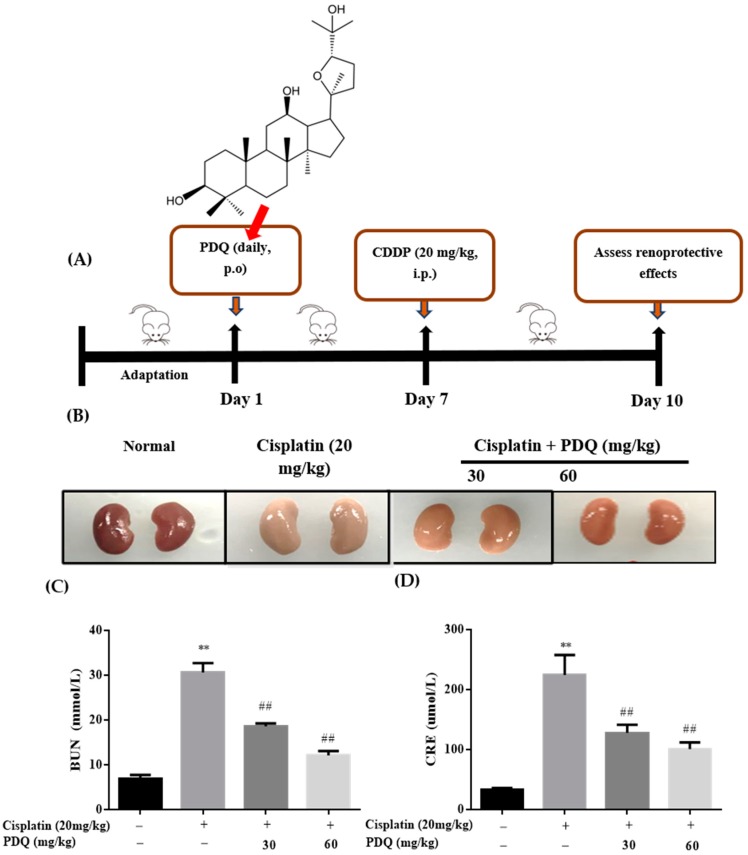
Experiment design (**A**); morphological change in kidney tissues (**B**); effects of PDQ on the levels of blood urea nitrogen (BUN) (**C**) and creatinine (CRE) (**D**). *n* = 8, ** *p* < 0.01 vs. normal group; ## *p* < 0.01 vs. cisplatin group.

Renal morphological changes in the mice from four groups are shown in Figure 1B. The biochemical parameters kidney index (KI), blood urea nitrogen (BUN), and creatinine (CRE) of the CDDP group were significantly increased, while ponderal growth and spleen index (SI) were markedly decreased compared to those of the normal group (*p* < 0.01). This result indicated that a rodent model of AKI was successfully reproduced [18]. 

Compared to the CDDP group, the levels of BUN and CRE in mice treated with PDQ (30 mg/kg, 60 mg/kg) were significantly reduced (*p* < 0.01) (Figure 1C,D), the increase in KI was significantly inhibited by PDQ (60 mg/kg) (*p* < 0.05), and abnormal decreases in SI were significantly reversed by PDQ (30 mg/kg, 60 mg/kg) (*p* < 0.05, *p* < 0.05) (Table 1).

### 2.2. PDQ Attenuates CDDP-Induced Histopathological Damage in Kidney

**Figure 2:**
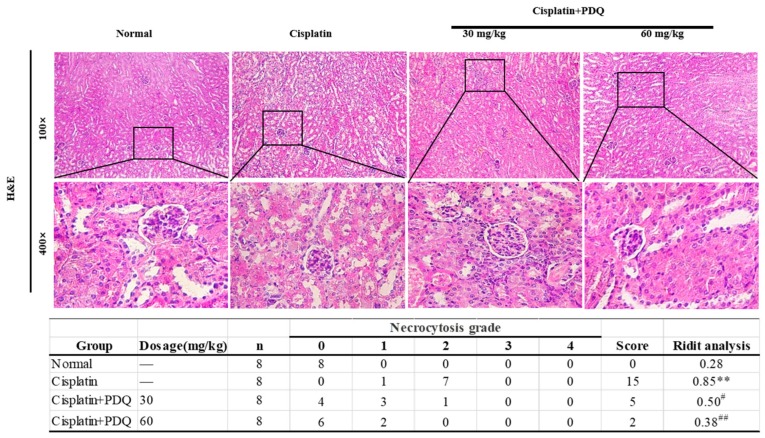
Histological examination of morphological changes in kidney tissues. Renal tissues were stained with hematoxylin and eosin (H&E) (100×, 400×), and the necrocytosis grade was assessed by ridit analysis. *n* = 8, ** *p* < 0.01 vs. normal group; # *p* < 0.05, ## *p* < 0.01 vs. cisplatin group.

Hematoxylin and eosin (H&E) staining and ridit analysis showed that cisplatin challenge led to distinguishing tubular necrosis and inflammatory infiltration in kidney tissues. However, PDQ induced the recovery of tubules to histological regularity and reduced inflammatory infiltrate cells and necrosis in kidney tissues (Figure 2). 

### 2.3. PDQ Reduces CDDP-Induced Tubular Cell Apoptosis in Kidney

**Figure 3:**
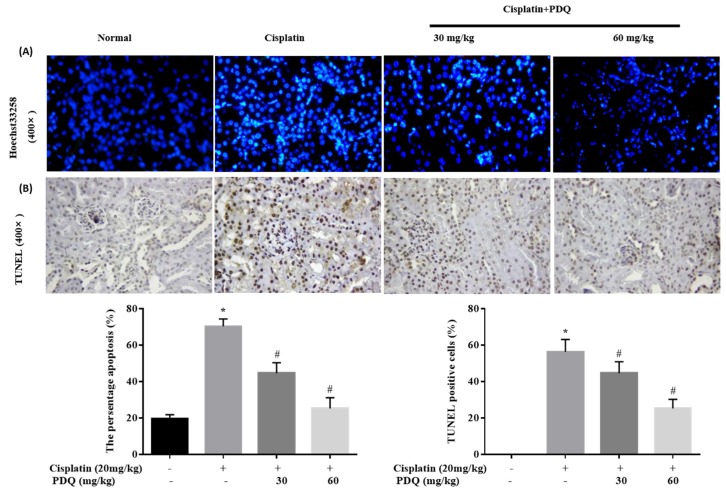
Histological examination of morphological changes in kidney tissues. Renal tissues stained with Hoechst 33258 (400×) (**A**) and TdT-mediated dUTP nick-end labeling (TUNEL) (400×) (**B**); renal tubular cell apoptosis and the presence of TUNEL-positive cells were measured by an image analyzer. *n* = 8, * *p* < 0.05 vs. normal group; # *p* < 0.05 vs. cisplatin group.

**Figure 4:**
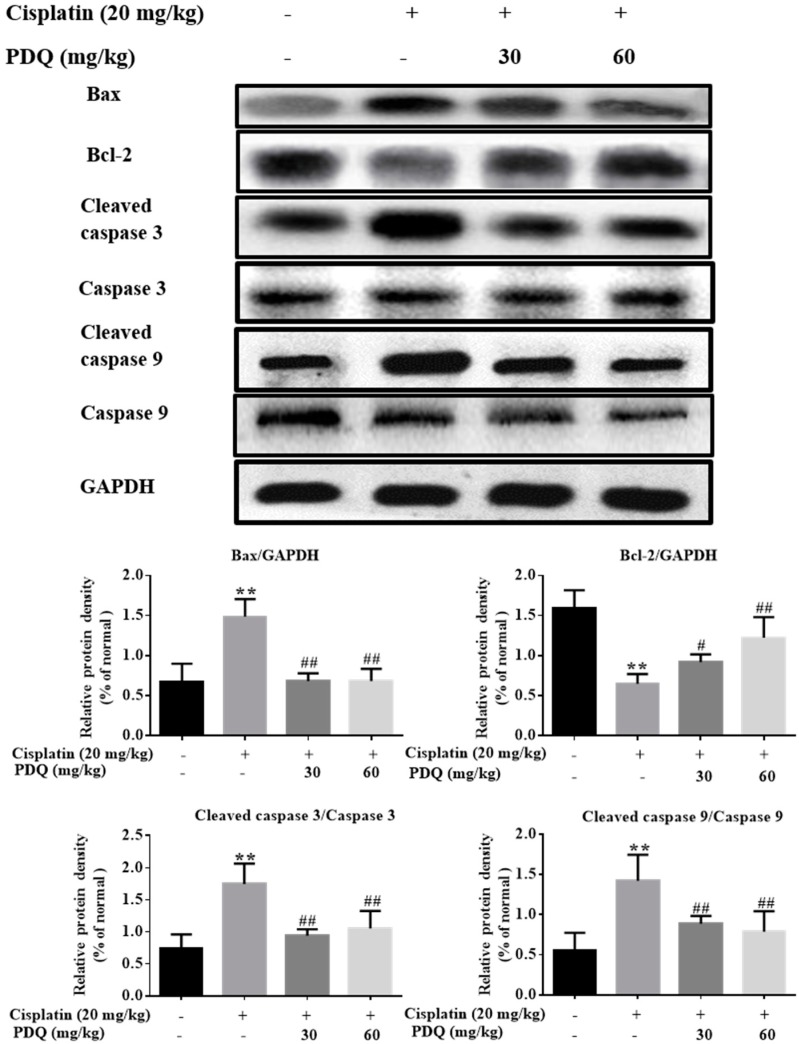
Effects of PDQ on the protein expression of Bax, Bcl-2, caspase-3, and caspase-9. *n* = 3, ** *p* < 0.01 vs. normal group; # *p* < 0.05, ## *p* < 0.01 vs. cisplatin group.

Apoptosis plays a key role in CDDP-induced renal damage [6,19]. The result of Hoechst 33258 staining showed that kidney sections in the CDDP group exhibited nuclear fragmentations and condensations that were quite higher compared to the normal group. However, round-shaped nuclei with homogeneous fluorescence intensity and regular contours were observed in the PDQ group. A similar tendency was observed in TdT-mediated dUTP nick-end labeling (TUNEL) staining: compared to the normal group, more TUNEL-positive stained cells were observed in the CDDP group, and PDQ significantly attenuated tubular cell apoptosis (Figure 3). Immunoblot analysis showed that CDDP challenge upregulated the protein levels of Bax, caspase 3, and caspase 9, and decreased the expression of Bcl-2. On the contrary, PDQ downregulated the expression of Bax, caspase 3, and caspase 9, and augmented the expression of Bcl-2, compared to the CDDP group (Figure 4). As Hoechst 33258 staining and TUNEL staining showed, PDQ at a dose of 60 mg/kg exhibited a dose-dependent beneficial effect by attenuating tubular cell apoptosis. Therefore, PDQ at a dose of 60 mg/kg was chosen as the cotreatment with CDDP for the antitumor study. 

### 2.4. PDQ Ameliorates CDDP-Induced Oxidative Stress in Kidney

**Figure 5:**
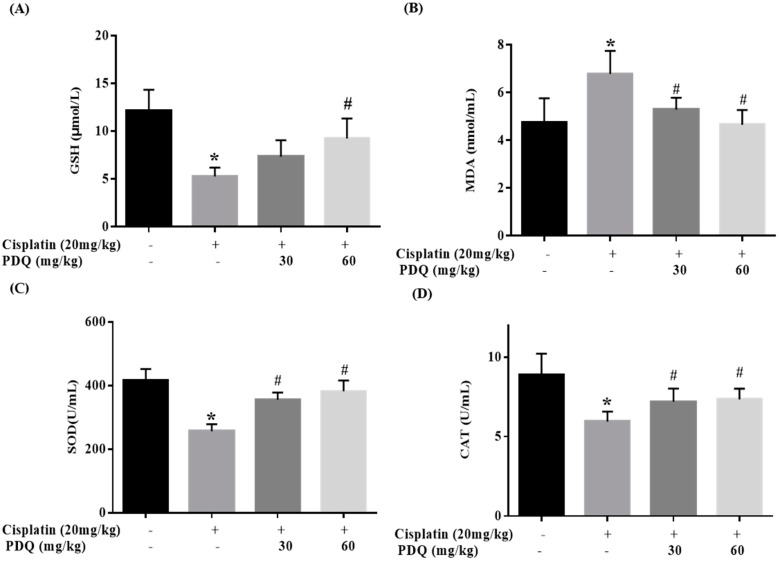
Effects of PDQ on the levels of glutathione (GSH) (**A**), malondialdehyde (MDA) (**B**), superoxide dismutase (SOD) (**C**), and catalase (CAT) (**D**) in cisplatin-induced acute kidney injury (AKI). All data are expressed as the mean ± S.D., *n* = 8. * *p* < 0.05 vs. control group; # *p* < 0.05 vs. cisplatin group.

**Figure 6:**
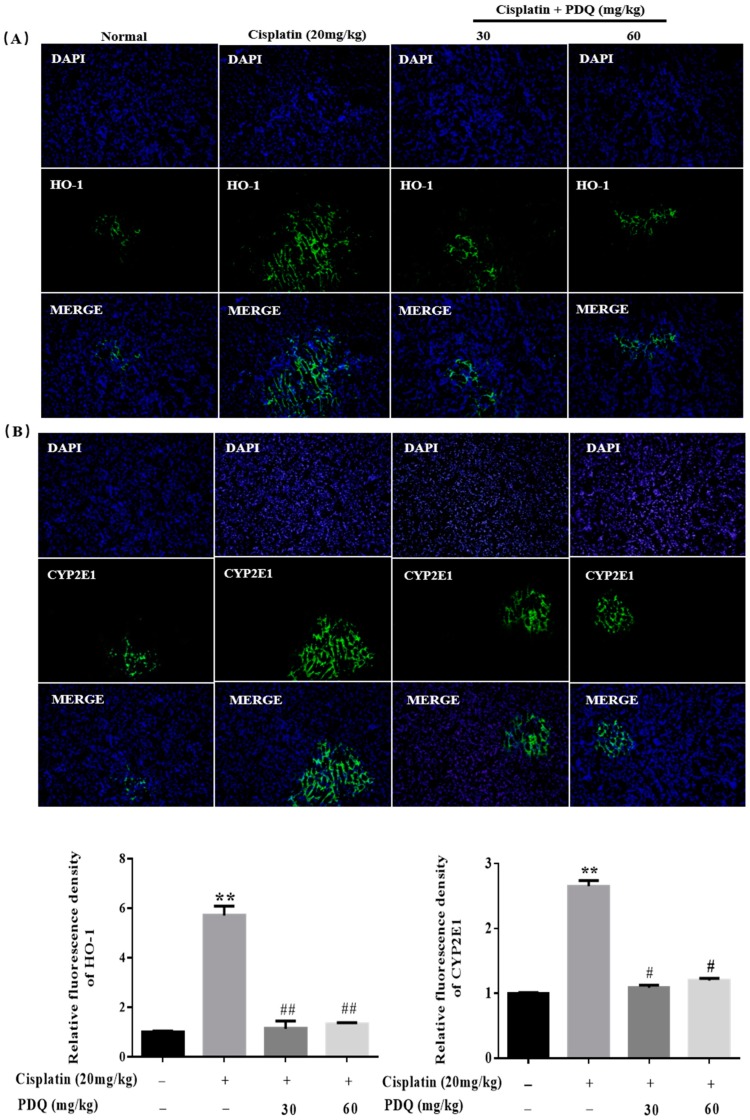
Pretreatment with PDQ inhibited the cisplatin (CDDP)-induced increase of heme oxygenase 1 (HO-1) (green) (**A**) and cytochrome P450 E1 (CYP2E1) (green) (**B**) in renal tubular epithelial cells. The expression levels of HO-1 and CYP2E1 were assessed by immunofluorescence staining (magnification, 200×). *n* = 8, ** *p* < 0.01 vs. control group; # *p* < 0.05, ## *p* < 0.01 vs. cisplatin group. DAPI (blue) was used as a nuclear counterstain.

**Figure 7:**
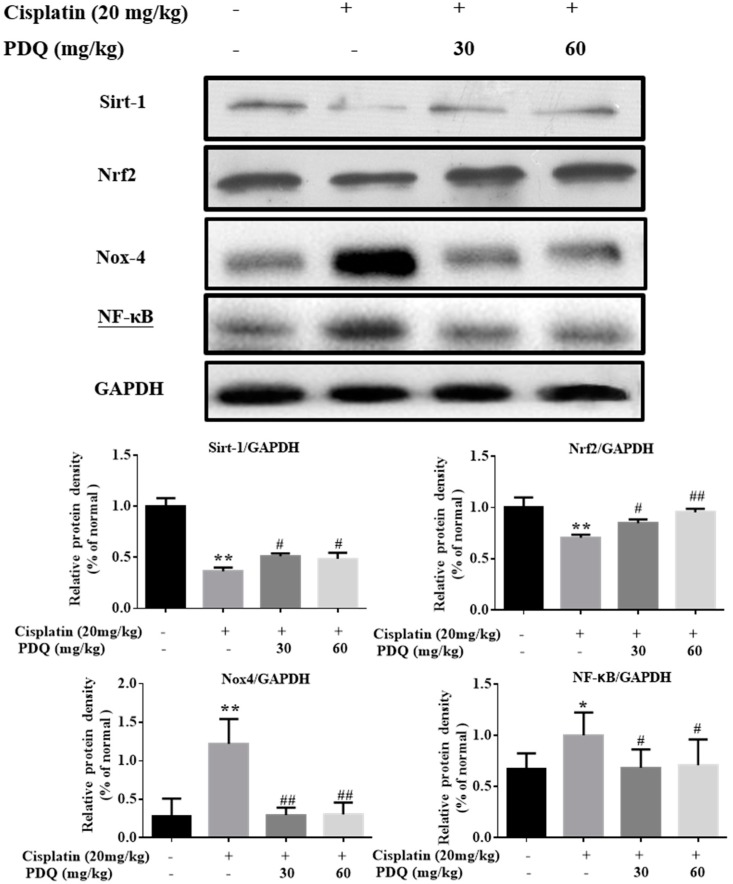
Effects of PDQ on the protein expression of Sirtuin-1 (Sirt-1), nuclear-related factor 2 (Nrf2), Nox-4, and nuclear factor-kappa B (NF-κB). *n* = 3, * *p* < 0.05, ** *p* < 0.01 vs. normal group; # *p* < 0.05, ## *p* < 0.01 vs. cisplatin group.

The level of malondialdehyde (MDA) was clearly increased, while the levels of glutathione (GSH), catalase (CAT), and superoxide dismutase (SOD) were markedly decreased in renal tissues after CDDP challenge (*p* < 0.05). Conversely, pretreatment with PDQ markedly reversed these changes (*p* < 0.05) (Figure 5). Immunofluorescence staining results showed that, compared to the normal group, heme oxygenase 1 (HO-1) and cytochrome P450 E1 (CYP2E1) in tubular cells were apparently increased in the kidney after CDDP challenge. However, the abnormal levels of HO-1 and CYP2E1 were clearly downregulated by PDQ (*p* < 0.05) (Figure 6). Immunoblot analysis showed that CDDP challenge decreased the expression of Sirt1 and Nrf2 and increased the expression of Nox-4, while PDQ reversed the changes in these proteins (Figure 7).

### 2.5. PDQ Suppresses CDDP-Induced Inflammation in Kidney

**Figure 8:**
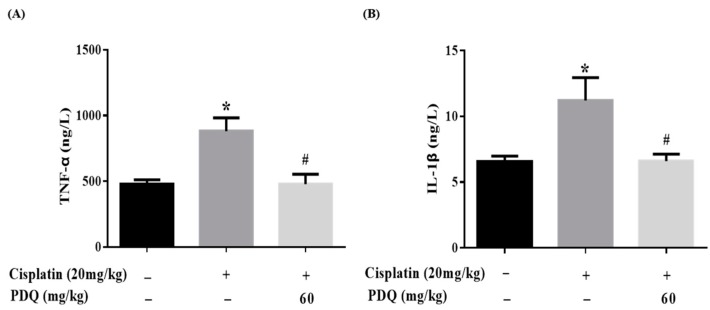
Effects of PDQ on the levels of inflammatory cytokines in cisplatin-induced nephrotoxicity. The levels of tumor necrosis factor-*α* (TNF-*α*) (**A**) and interleukin-1*β* (IL-1*β*) (**B**) in kidney tissues were determined by ELISA kits. All data are expressed as mean ± S.D., *n* = 8. * *p* < 0.05 vs. control group; # *p* < 0.05 vs. cisplatin group.

**Figure 9:**
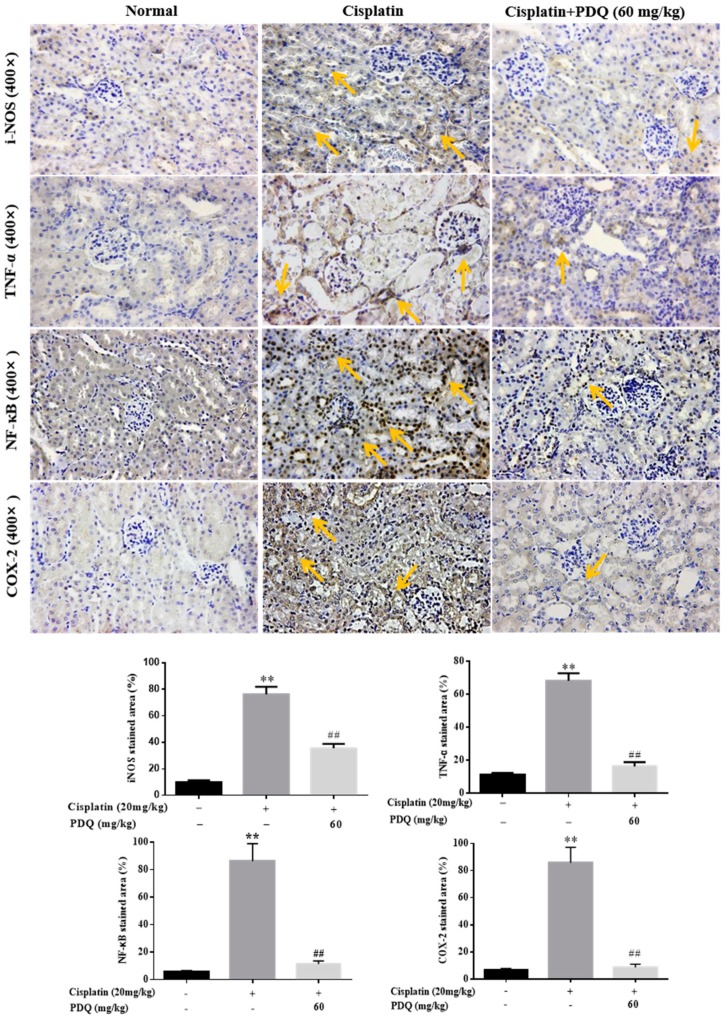
Effects of PDQ on the expression of inducible nitric oxide synthase (i-NOS), TNF-*α*, NF-κB, and cyclooxygenase-2 (COX-2); (400×). Protein expression was examined by immunohistochemistry; arrows show positive protein expression in renal tubular epithelial cells. *n* = 8, ** *p* < 0.01 vs. control group; ## *p* < 0.01 vs. cisplatin group.

Inflammation is accompanied by oxidative stress in CDDP-induced AKI [19]. In the CDDP group, the levels of the proinflammatory cytokines TNF-*α* and IL-1*β* in serum were elevated dramatically (*p* < 0.05) compared to the normal group, but these changes were reversed by PDQ (Figure 8). As shown by immunohistochemical analysis, quite low levels of these proteins were detected in the normal group. After CDDP challenge, highly positively expressed TNF-α, inducible nitric oxide synthase (iNOS), and cyclooxygenase-2 (COX-2) in cytoplasm and NF-κB in the nucleus were detected in the tubular cells, whereas administration with PDQ dramatically downregulated the rates of positive expression of TNF-*α*, NF-κB, iNOS, and COX-2 in renal tissues (Figure 9). Immunoblot analysis showed that CDDP challenge increased the expression of NF-κB, while PDQ downregulated NF-κB expression (Figure 7). 

### 2.6. PDQ Does Not Compromise the In Vivo Antitumor Activity of CDDP

**Figure 10:**
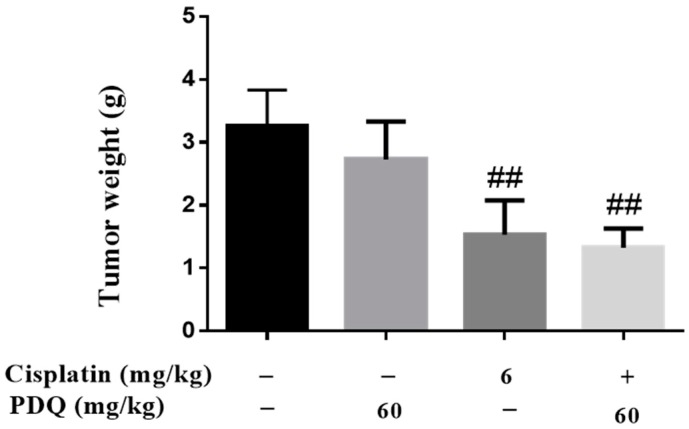
Effects of PDQ on the antitumor activity of cisplatin against Lewis lung cancer xenograft tumors in C57BL/6 mice. *n* = 10, ## *p* < 0.01 vs. model control group.

As shown in Figure 10, treatment with PDQ exhibited no significant antitumor effect, but cotreatment with PDQ (60 mg/kg) augmented the growth-inhibiting properties of CDDP.

## 3. Discussion

Cisplatin is one of the most effective antitumor drugs in clinical chemotherapy, but cisplatin-induced nephrotoxicity causes many patients to suffer from severe renal function decline [15]. Previous studies have shown that apoptosis, inflammation, and oxidative stress are involved in CDDP-induced nephrotoxicity [6,19]. In this study, we report, for the first time, the protective effect of PDQ against cisplatin-induced nephrotoxicity without compromising its antitumor activity.

Three days after CDDP challenge, we saw a sharp rise in the kidney index and serum BUN and CRE—the most sensitive renal function parameters—and a decrease in body weight and the spleen index in the model group compared to the normal group. However, the above renal dysfunction indicators and the decreased immune function were improved by PDQ treatment.

Oxidative stress is an important factor in the pathogenesis of CDDP-induced kidney toxicity [15,20]. Sirt1 exerts potent antioxidant effects by enhancing the transcriptional activity of Nrf2, an important antioxidant transcription factor that is protective against oxidative stress [21]. Nox4 is known as the predominant form of the Nox family members, and it is highly expressed in the kidney and plays an important role in renal oxidative stress and kidney injury [22]. In kidney tissues, CDDP challenge caused markedly decreased protein levels of Sirt1 and Nrf2 and a significant overexpression of Nox4, resulting in GSH depletion, reduced levels of the antioxidant enzymes SOD and CAT, the overexpression of the drug-metabolizing enzyme CYP2E1 and the phase II antioxidant enzyme HO-1, and higher levels of MDA, the end-product of lipid hydroperoxidation—these are the indicators of oxidative stress [19,23]. However, PDQ effectively reversed these changes so that these proteins returned to relatively normal levels. Therefore, the suppression of oxidative stress may be one mechanism by which PDQ protects against CDDP-induced kidney injury.

Cisplatin exposure can promote the release of the proinflammatory cytokines TNF-*α* and IL-1*β* [15] and upregulate NF-κB expression in kidney tissues [24]. An increased level of COX-2, the inducible form of COX, has been often found in kidney tissues with cisplatin-induced inflammation [25]. Also, the obvious expression of iNOS was found to be accompanied by COX-2 at sites of inflammation, leading to the aggravation of the inflammatory reaction [26]. In our work, PDQ significantly downregulated serum TNF-*α* and IL-1*β* and decreased the protein expression of iNOS, TNF-*α*, NF-κB, and COX-2, all of which aggressively increased in kidney tissues after CDDP challenge. In a word, PDQ inhibited the inflammatory reaction in CDDP-induced AKI.

Apoptosis is a predominant cellular activity that is responsible for kidney pathogenesis in CDDP-induced AKI [27]. Various proteins, such as Fas receptors and caspases, promote apoptosis, while some proteins in the Bcl-2 family, including Bcl-2, inhibit apoptosis. Bax, known as a pro-apoptosis regulator, is a protein encoded by the *BAX* gene that resides in the cytosol and translocates to mitochondria upon apoptosis induction [19]. A decline in Bcl-2 expression causes the release of the apoptosome [9]. The apoptosome turns pro-caspase 9 into its active form, cleaved caspase-9, which then activates the effector caspase-3 and triggers the execution of tubular cell apoptosis [28,29]. We observed that PDQ significantly attenuated CDDP-induced renal apoptosis and suppressed the protein expression of Bax, caspase-9, and caspase-3, and upregulated the level of Bcl-2, which illustrates that the caspase-mediated pathway may be another relevant mechanism underlying the protective actions of PDQ against CDDP-induced AKI.

## 4. Materials and Methods

### 4.1. Chemicals and Reagents

PDQ (batch no. 20171015, purity > 98.0%) was prepared in the School of Pharmaceutical Sciences of Jilin University (Changchun, China). Cisplatin (CDDP, purity > 99.0%) was obtained from Sigma Chemicals (St. Louis, MO, USA). Commercial assay kits for hematoxylin and eosin (H&E) dye, BUN, CRE, MDA, GSH, SOD, and CAT were all provided by Nanjing Jiancheng Bioengineering Research Institute (Nanjing, China). TNF-*α* and IL-1*β* ELISA kits were purchased from R&D systems (Minneapolis, MN, USA). The Hoechst 33258 dye kit was bought from Shanghai Beyotime Co., Ltd. (Shanghai, China). The TUNEL staining kit was bought from Roche Applied Science (Indianapolis, IN, USA). The two-site immunohistochemistry assays kits, SABC-CYP2E1 and SABC-HO-1 immunofluorescence staining kits, were provided by BOSTER Biological Technology Co., Ltd. (Wuhan, China). Antibodies for Western blots and immunohistochemistry were provided by Cell Signaling Technology (Danvers, MA, USA). Other reagents and chemicals were from Beijing Chemical Works (Beijing, China)

### 4.2. Animals and Experimental Protocol

SPF grade ICR mice (male, 22–25 g) and C57BL/6 mice (male, 18–22 g) from Changchun Yisi Experimental Animal Co. Ltd. (Certificate of Quality No. of SCXK (JI) 2016-003, Changchun, China) were raised under a 12 h light/dark cycle, relative humidity of 60 ± 5%, and 25 ± 2 °C for 1 week to acclimatize to the new conditions before the experiment. The experiments were performed in strict accordance with the Regulations of Experimental Animal Administration from the Ministry of Science and Technology of China. All experimental procedures in this work were approved by the Ethical Committee for Laboratory Animals at Jilin Agricultural University (Permit No.: ECLA-JLAU-17065). 

After adaptive breeding for 1 week, ICR mice were randomly divided into four groups (*n* = 8): (1) normal, (2) CDDP (20 mg/kg), (3) CDDP + PDQ (30 mg/kg), (4) CDDP + PDQ (60 mg/kg). PDQ was suspended in 0.05% carboxymethylcellulose sodium (CMC-Na); CDDP was dissolved in 0.9% NaCl. The mice in the treatment groups were gavaged (P.O) with PDQ for 10 consecutive days; normal and CDDP mice were treated with 0.05% CMC-Na in the same way. On the 7th day, animals in the cisplatin control and PDQ-treated groups were given a single dose of CDDP (20 mg/kg; i.p.) after 30 min from the last administration of PDQ to induce AKI in mice; the normal group received one dose of 0.9% NaCl (i.p.). Mice were fasted (free access to water) before administration with CDDP and dissection. Three days after cisplatin injection, the whole blood was collected from the orbit and mice were sacrificed by cervical vertebra dislocation. Serum was separated from the blood by centrifugation (3500 rpm, 15 min, 4 °C) and stored at −20 °C. Spleens and kidneys were rapidly collected for subsequent assays.

### 4.3. Assessment of Biochemical Parameters

The serum BUN and CRE levels were measured according to the manufacturer’s protocol. The right kidney was homogenized in 0.9% NaCl and centrifuged (10,000 rpm, 10 min, 4 °C). The supernatants were separated and the levels of MDA, GSH, SOD, and CAT in kidney tissues were measured according to the manufacturer’s protocol.

### 4.4. Measurement of Serum TNF-α and IL-1β Levels

Serum TNF-*α* and IL-1*β* were measured by ELISA kits according to the manufacturer’s protocol. 

### 4.5. Histological Assays 

The kidney samples were immobilized with 10% formalin before paraffin embedding and sectioned into sections with a thickness of 5 μm. The slices were stained with H&E dye kits according to the manufacturer’s protocol and photographed using light microscopy (Leica DM750, Solms, Germany) and scored based on the percentage of epithelial necrosis in the cortical tubules: 0: no damage; 1: <10%; 2: 10–25%; 3: 26–75%; 4: >75%. 

### 4.6. Hoechst 33258 and TUNEL Staining

The paraffin slices from each group were stained with Hoechst 33258 solution. The stained nuclei were photographed using a fluorescence microscope (Leica TCS SP8, Solms, Germany) under ultraviolet excitation and quantified by Image-Pro plus 6.0 (Media Cybernetics, Rockville, MD, USA). TUNEL staining was performed with TUNEL apoptosis detection kits and photographed with a light microscope (Leica DM750, Solms, Germany). 

### 4.7. Immunohistochemistry (IHC) and Immunofluorescence Analysis

For IHC staining, the paraffin slices were incubated with primary antibodies to NF-κB (1:200), TNF-*α* (1:200), iNOS (1:200), and COX-2 (1:200), respectively, overnight at 4 °C in a humidified chamber, followed by incubation with horseradish peroxidase-conjugated secondary antibodies for 1 h at 37 °C. A brownish yellow color in the cytoplasm or nucleus was regarded as positive cellular staining. The image was photographed using a light microscope and the intensity of cells’ positive expression was quantified by Image-Pro Plus 6.0. For immunofluorescence staining, the kidney slices were incubated with primary antibodies to CYP2E1 (1:200) and HO-1 (1:200), respectively, overnight at 4 °C in a humidified chamber, followed by incubation with DyLight 488-labeled secondary antibodies for 1 h at 37 °C. DAPI (4,6-diamidino-2-phenylindole) was used for nuclear staining. The image was photographed with a fluorescence microscope (Leica TCS SP8, Solms, Germany) and the intensity of the immunofluorescence was quantified by Image-Pro Plus 6.0.

### 4.8. Western Blotting Analysis 

Kidney tissues were lysed using RIPA buffer, and the proteins (50 µg/lane) were then electrophoresed on 12% SDS polyacrylamide gels and transferred to a PVDF membrane. After blocking for 2 h, the membrane was incubated with primary antibodies overnight at 4 °C [26]. Next, the membranes were incubated for 1 h with secondary antibodies at 37 °C. Finally, the signals were detected using an ECL substrate. The intensity of the bands was analyzed with Image-Pro Plus 6.0 and the intensity values were expressed as the relative protein expression normalized to GAPDH.

### 4.9. Establishment of Xenograft Tumor Model 

Lewis lung tumors were isolated from donor C57BL/6 mice and implanted in the dorsum of recipient C57BL/6 mice by intracutaneous injection. Then, the mice were randomly assigned into four groups (*n* = 10): (1) model control group: 0.9% NaCl (i.p., one dose on day 4) and CMC-Na (P.O, 10 days); (2) CDDP group: 6 mg/kg CDDP (i.p., one dose on day 4) and CMC-Na (P.O, 10 days); (3) PDQ group: 0.9% NaCl (i.p., one dose on day 4) and 60 mg/kg PDQ (P.O, 10 days); (4) PDQ + CDDP group: 6 mg/kg CDDP (i.p., one dose on day 4) and 60 mg/kg PDQ (P.O, 10 days). On day 11, mice were sacrificed, and tumors were separated and weighted.

### 4.10. Statistical Analysis

Data were analyzed with GraphPad Prism 6.0 software (CA, USA) and are presented as the mean ± S.D. Statistical significance was calculated with one-way analysis of variance (ANOVA), and *p*-value < 0.05 was considered significant.

## 5. Conclusions

This study demonstrates that PDQ protected renal cells against CDDP-induced AKI by a mechanism involving Sirt1/NF-κB and the caspase signaling pathway. In summary, pretreatment with PDQ evidently restored levels of BUN and CRE, reduced CDDP-induced tubular cell apoptosis, recovered the impaired antioxidant system—characterized by decreasing the CDDP-induced overexpression of HO-1 and CYP2E1—and alleviated inflammation, which manifested as a decrease in the levels of proinflammatory cytokines and inflammatory mediators, including IL-1*β* and TNF-*α*. Importantly, cotreatment with PDQ did not attenuate the antitumor activity of CDDP. This report provides new insights for further utilization of PDQ. 

## Figures and Tables

**Table 1:** Effects of PDQ (pseudoginsengenin of diol derivatives quest) on body weight change, kidney index, and spleen index in cisplatin-induced acute kidney injury.

Groups	Dosage (mg/kg)	Body Weights (g)		Kidney Index (mg/g)	Spleen Index (mg/g)
		Initial	Final		
Normal	-	30.37 ± 2.44	32.07 ± 1.51	16.25 ± 1.43	4.17 ± 0.56
Cisplatin	-	29.28 ± 1.57	27.41 ± 2.04	21.34 ± 1.96 **	1.71 ± 0.30 **
PDQ	30	31.32 ± 2.19	29.16 ± 2.54	19.28 ± 0.52	2.24 ± 0.25 ^#^
PDQ	60	31.44 ± 1.72	30.42 ± 1.73	18.03 ± 1.67 ^#^	2.47 ± 0.23 ^#^

*n* = 8, ** *p* < 0.01 vs. normal group; # *p* < 0.05 vs. cisplatin group.
